# Novel Application of Balloon Tamponade in Management of Acute Lower Gastrointestinal Hemorrhage

**DOI:** 10.5811/cpcem.2019.3.41772

**Published:** 2019-04-22

**Authors:** Michael M. Neeki, Vikram Raj, Benjamin Archambeau, Sarkis Arabian, Farabi Hussain

**Affiliations:** * Arrowhead Regional Medical Center, Department of Emergency Medicine, Colton, California; † Arrowhead Regional Medical Center, Department of Internal Medicine, Colton, California; ‡ Arrowhead Regional Medical Center, Department of Surgery, Colton, California

## Abstract

We present a case of acute lower gastrointestinal (GI) bleeding in the emergency department, in which specialists were not emergently available to render their support. A quick intervention using balloon tamponade technique with a Minnesota tube helped stabilize the patient until intensive care, gastroenterology, and surgical specialists could intervene. We also review previous cases from the literature in which a balloon tamponade method was used to control GI hemorrhage. Our novel application of the Minnesota tube is important for emergency physicians to consider for cases of acute lower GI bleeding, particularly in emergent presentations when specialists are not readily available in-hospital.

## INTRODUCTION

Lower gastrointestinal (GI) hemorrhage is defined as bleeding that arises from the GI tract at any site distal to the ligament of Treitz, which connects at the duodenojejunal flexure.^1^ The incidence of lower GI bleeding is reported to be from 20–36 per 100,000 persons.^2^,^3^ This pathology accounts for approximately 1–2% of all acute hospital admissions with 5–12% of cases resulting in mortality.^1^ Of these cases, nearly 95% are due to bleeding from the colon, whereas other reports estimate that 10–25% of cases are due to small bowel bleeding.^4^–^6^ Due to challenges in emergent localization of the sources of lower GI hemorrhage and effective treatment, acute lower GI hemorrhage remains a frustrating problem for both the treatment team and patient, especially when standard treatments fail to achieve hemostasis.^1^

**Image 1:**
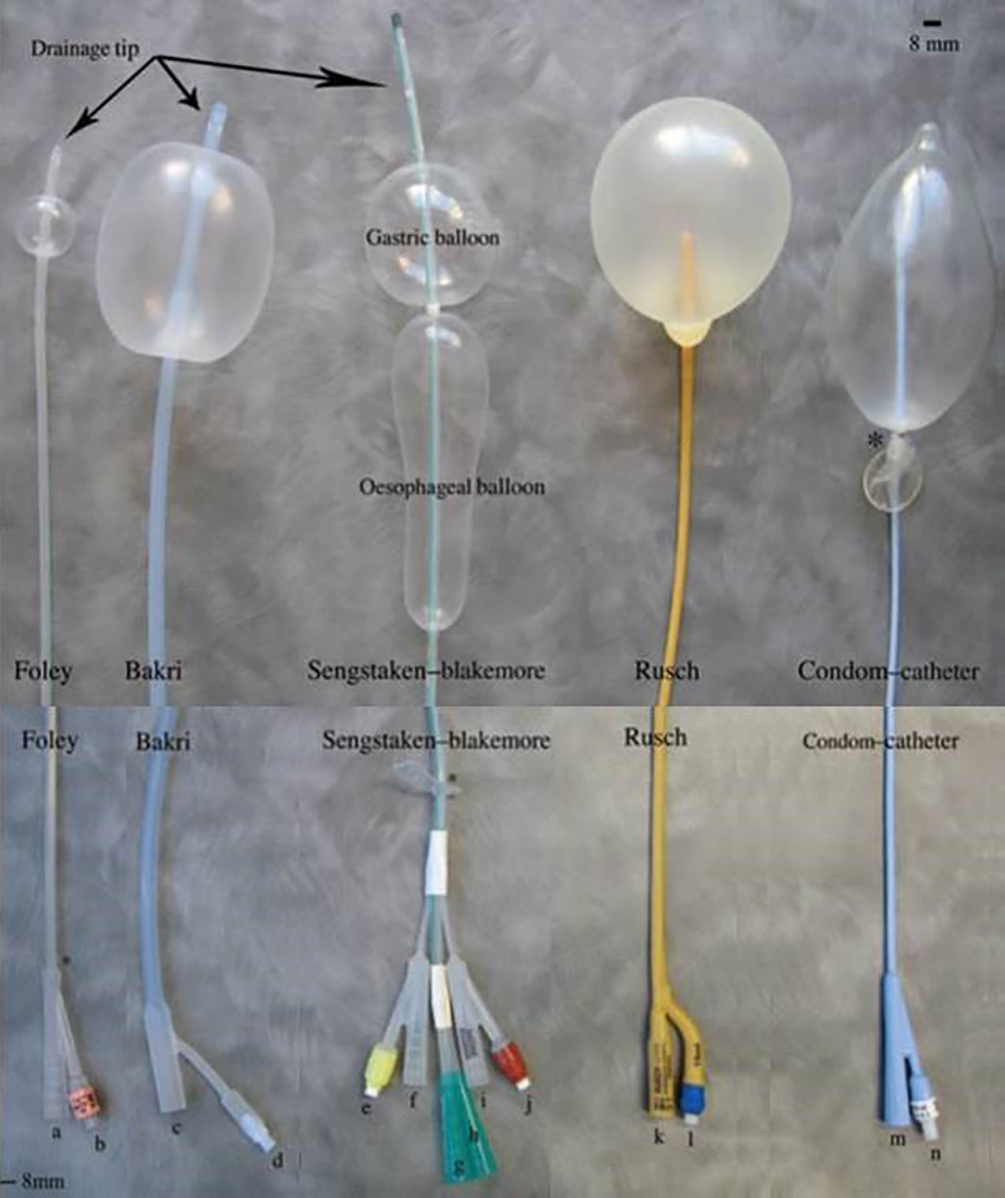
A variety of balloon tamponade devices showing the distal end with maximal inflation (top) and the proximal end (bottom).^10^

Acute lower GI hemorrhage can become life threatening if not diagnosed quickly and properly managed. Previous studies have explored the use of balloon tamponades in treatment of acute hemorrhage in fields such as obstetrics and gynecology, prehospital traumatic injury, and upper GI hemorrhage.^7^–^16^ Investigators have used various balloon tamponade devices (Image 1) for a range of conditions, including penetrating neck trauma, postoperative rectal hemorrhage, postpartum hemorrhage, and various variceal bleeds.^7^–^16^

**Image 2:**
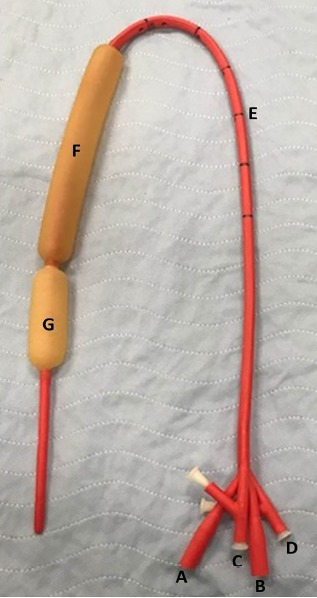
Minnesota tube with labeled components: A) gastric aspiration port; B) esophagus aspiration port; C) gastric balloon port; D) esophagus balloon port; E) length marks for placement; F) esophageal balloon; G) gastric balloon.

In particular, several balloon tamponade devices were specifically designed as temporary measures for upper GI hemorrhage, particularly in patients with shock indices greater than 1.3.^17^,^18^ For example, studies involving Sengstaken-Blakemore tube utilization in upper GI hemorrhage demonstrated successful hemostasis of variceal hemorrhage in up to 94% of cases, although 38% of patients experienced rebleeding and 10% suffered major complications such as pressure ulceration and necrosis.^8^ Similarly, the Minnesota tube (Image 2), which is the Sengstaken-Blakemore tube’s successor, is a 4-lumen esophagogastric balloon tamponade device indicated for the treatment of gastric and esophageal variceal exsanguination.^19^ Previous studies have demonstrated the utility of the Minnesota tube in upper GI bleeding with a case series of 100 patients showing that balloon tamponade was successful in achieving hemostasis in 61% of cases, while other studies have indicated a success rate of approximately 50%.^1^,^20^,^21^ As these studies suggest, balloon tamponade devices are viable tools in a wide range of situations involving hemorrhage, acting as a bridge until definitive interventions are available.

We present a unique case of acute massive lower GI hemorrhage in the emergency department (ED), where a balloon tamponade technique using a Minnesota tube was successful in achieving hemostasis at a critical time when no definitive intervention was available. The goal of this case report is to alert emergency physicians to the unique utility of balloon tamponade in the treatment of non-localized, lower GI hemorrhage.

## CASE REPORT

A 76-year-old, wheelchair-bound male with history of hypertension, chronic kidney disease, and a prior thoracic and abdominal aortic repair, presented to the ED with an altered level of consciousness and was assessed with a Glasgow Coma Scale of nine (Eye 2, Verbal 3, Motor 4) and generalized weakness after examination. Emergency medical services noted bright red blood during the transfer, and upon arrival to the ED the patient presented hypotensive at 74/45 milligrams of mercury (mmHg), a heart rate of 102 beats per minute, a shock index of 1.4, a temperature of 97° Fahrenheit, 94% saturation breathing room air, and a respiratory rate of 18 breaths per minute. Given the patient’s hypotensive status, we suspected the altered level of consciousness to be due to the acute hemorrhage.

Initially, the bleeding seemed to be minimal, although his hemoglobin, hematocrit, and platelet counts were notably low, with hemoglobin at 6.6 grams per deciliter, hematocrit at 20.7%, and platelet count of 104,000 units per liter. The patient was intubated using rapid sequence intubation to protect his airway, and two large antecubital intravenous (IV) lines and later a right internal jugular vein central venous catheter were placed to administer blood products and medications. Subsequently, the massive transfusion protocol was activated with immediate transfusion with two units of packed red blood cells (PRBCs) in addition to one gram IV tranexamic acid over 10 minutes and an additional one gram infused over the next eight hours. As the patient presented at late evening hours, specialists such as GI and interventional radiology teams were unavailable in-house. However, the gastroenterology and interventional radiology specialist teams were remotely consulted by telephone in the management of the patient. In addition, the patient’s condition was too unstable to allow him to be transferred for imaging studies.

**CPC-EM Capsule**

What do we already know about this clinical entity?

Emergent lower gastrointestinal (GI) hemorrhage is a potentially fatal event with a variable presentation.

What makes this presentation of disease reportable?

Patients with lower GI hemorrhage often present to emergency departments. Unfortunately, access to tools and specialists is widely variable and may affect patient outcomes.

What is the major learning point?

Timely treatment of acute onset of lower GI hemorrhage is essential. Balloon tamponade devices may present an additional tool to improve mortality and morbidity outcomes.

How might this improve emergency medicine practice?

Balloon tamponade devices may present an opportunity to control bleeding and stabilize the patient until definitive treatment, but only in appropriate cases and with close observation.

**Image 3:**
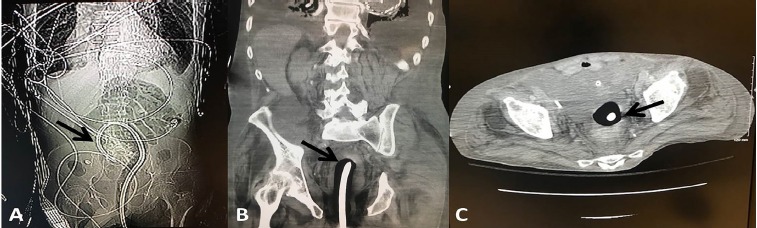
Radiologic images of balloon tamponade placement with arrows indicating the inflated Minnesota tube: A) radiograph showing the full placement; B) computed tomography (CT) of coronal plane showing placement in the descending colon; C) CT of axial plane showing placement in the colon.

As the initial transfusion started, the patient began to bleed profusely via his rectum. The treatment team was unable to localize the source of bleeding, and direct visualization using an anoscope was not feasible due to acute perfuse hemorrhaging from the rectum. Attempts at applying direct, intrarectal pressure with gauze were unsuccessful. Subsequently, a Foley balloon accompanied with direct pressure to the site also proved ineffective. Based on the patient’s past history of constipation along with abdominal aortic aneurysm repair, a colonic tear along with a possible aortoenteric fistula was suspected to be the cause of the perfuse bright red bleeding. Since other standard and alternative interventions were exhausted, a decision was made to use a Minnesota tube, a balloon tamponade device traditionally used for upper GI bleeding, transrectally in an attempt to control the bleeding. The gastric balloon was initially inflated with 200 milliliters (mL) of air without resolution of bleeding, at which point the esophageal port was inflated with 300 mL of air to a pressure of 40 mmHg, which ultimately resulted in adequate hemostasis. The patient was then resuscitated with continuous transfusion of blood products and stabilized in the ED prior to undergoing a diagnostic computed tomography (CT) of abdomen and pelvis in an attempt to localize the source of the hemorrhage (Image 3). Following the CT, which showed no evidence of acute aortioenteric pathology or acute bleeding within colonic mucosa, the patient was transported to the intensive care unit, where he did not experience any further episodes of rectal bleeding. Over the course of the night, he received an additional six units of PRBCs, two units of plasma, two units of platelets, and two units of cryoprecipitate. The following morning, the gastroenterology team removed the Minnesota tube prior to performing a flexible sigmoidoscopy. During the flexible sigmoidoscopy, a circumferential, ulcerated mucosa at the dentate line was identified as the source of the profuse bleeding and was cauterized. The patient was then extubated, observed overnight without complications, and finally discharged 48 hours post-arrival to an extended care facility in a stable condition.

## DISCUSSION

In treating cases of GI hemorrhage with balloon tamponade, differences in upper and lower GI tissue should be considered. Upper GI mucosa epithelium is keratinized and possesses protective mechanisms that contribute to its ability to withstand harsh, acidic environments.^22^ As a result, conditions that compromise the protective mucous layer, such as peptic ulcer disease, may result in bleeding.^23^,^24^ In contrast, the anal canal tissue is composed of columnar epithelial cells, which thinly overlie the hemorrhoidal venous plexuses, contributing to the risk of massive hemorrhage.^25^ Etiology for lower GI hemorrhage is predominately caused by colonic diverticulosis, followed by internal hemorrhoids, with ischemic colitis and post-polypectomy being an increasing minority of causes.^3^ These structural differences of lower GI mucosa and the increased risk of mechanical iatrogenic complications may warrant additional caution when considering balloon tamponade in treating lower GI hemorrhage.

Potential complications from the use of balloon tamponade may include ulceration and pressure necrosis resulting from compression of the visceral wall via increased pressure of the balloon tamponade device, which may ultimately cause perforation, especially in cases of excessive or prolonged inflation.^7^,^26^ To avoid potential complications, the following recommendations should be considered: sub-maximal balloon inflation for a maximum of 12 hours, careful monitoring, and gradual deflation in the case of visceral pain.^7^ In our case we used a Minnesota tube, originally designed for esophageal bleeds, in a situation involving a lower GI bleed. Given the structural differences between upper and lower GI mucosal structure, these aforementioned precautions may require more conservative adjustment. The propensity for lower GI bleeds to result from physical factors may warrant lower levels of inflation than the maximal recommended pressure of 45 mmHg for esophageal bleeds.^27^ Other precautions should include a reduced maximum duration of balloon inflation and a more gradual deflation.

Balloon tamponade has rarely been employed for lower GI hemorrhage, and previous successful cases have mostly been anecdotal via case reports (Table). Our use of a Minnesota tube in a case of lower GI hemorrhage provides additional evidence that balloon tamponade may be an effective and accessible alternative to establish hemostasis in a variety of hemorrhagic situations and provide necessary stabilization during the initial phase of resuscitation prior to definitive intervention.

## CONCLUSION

Our case provides emergency physicians with a critical example of an alternative method of stabilization of a non-localized, massive lower GI hemorrhage using balloon tamponade when all other alternative methods failed to achieve hemostasis. This novel case may provide a basis for both further research and support for ED providers in the future to consider non-standard utilization of readily available resources for emergent cases of bleeding. It is also important to consider the possible complications of such a procedure, although none occurred in this case. While there is precedent for this treatment modality, the risks and benefits of its use should be weighed, and all avenues of standard treatment should first be exhausted.

## Figures and Tables

**Table:** Previously published case reports detailing subject’s age and gender, location of the hemorrhage, presentation/case details, type of balloon tamponade used, and patient outcome.

Author	Subject	Location	Presentation/case details	Type of balloon	Outcome
Braley et al. 2002^14^	54M	Lower GI (presacral)	Lower GI bleeding after removal of GI stromal tumor	Breast implant sizer	Adequate hemostasis until surgery performed
McGuinness et al. 2004^7^	65M	Lower GI (transanal)	Lower GI bleeding after removal of rectal adenoma	Minnesota tube w/gauze	No further bleeding
Marshall et al. 2007^9^	54M	Lower GI (iliorectal anastomosis)	Bleeding after formation of a ileorectal anastomosis following a blood transfusion	Minnesota tube	No complications
Su Min Cho, 2006^28^	51M	Lower GI (rectal varices)	Bleeding 24 hours after polypectomy, and then rebleed after laparotomy	Minnesota tube	No further bleeding

*M,* Male; *GI,* gastrointestinal.
